# Effect of Mean Platelet Volume to Platelet Count Ratio on Mortality in Peritoneal Dialysis

**DOI:** 10.1155/2022/6922809

**Published:** 2022-11-10

**Authors:** Jiasi Chen, Zhong Zhong, Jianbo Li, Jianwen Yu, Yi Yu, Bin Li, Haiping Mao, Zhijian Li

**Affiliations:** ^1^Department of Nephrology, The First Affiliated Hospital, Sun Yat-sen University, Guangzhou 510080, China; ^2^NHC Key Laboratory of Clinical Nephrology (Sun Yat-sen University) and Guangdong Provincial Key Laboratory of Nephrology, Guangzhou 510080, China; ^3^Clinical Trials Unit, The First Affiliated Hospital, Sun Yat-sen University, Guangzhou 510080, China

## Abstract

**Background and Aims:**

Mean platelet volume to platelet count ratio (MPV/PC) has been found to be an independent risk factor for mortality in various diseases, including cardiovascular disease, cancer, and hemodialysis. We aimed to evaluate the association between MPV/PC and all-cause and cardiovascular (CV) mortality in peritoneal dialysis (PD) patients.

**Methods and Results:**

We conducted a retrospective cohort study at a single center and enrolled 1473 PD patients who were catheterized at our PD center from January 1, 2006, to December 31, 2013. All patients were divided into four groups according to the quartiles of baseline MPV/PC levels and followed up until December 31, 2018. A total of 453 patients died, and 221 deaths were caused by cardiovascular disease during a median follow-up time of 48.0 (21.9-82.2) months. There was a significant interaction by age of association between MPV/PC level and all-cause mortality (*P* = 0.009), and multivariate Cox regression analysis showed that higher MPV/PC level was related to a decreased risk of all-cause and CV mortality in PD patients aged < 60 years (HR = 0.62, 95%CI = 0.40 − 0.96, *P* = 0.032; HR = 0.49, 95%CI = 0.26 − 0.93, *P* = 0.029, respectively), rather than in patients aged ≥ 60 years (HR = 1.37, 95%CI = 0.84 − 2.22, *P* = 0.208; HR = 1.50, 95%CI = 0.77 − 2.92, *P* = 0.237, respectively).

**Conclusion:**

Our results indicated that low MPV/PC level was an independent risk factor for all-cause and CV mortality in PD patients aged less than 60 years.

## 1. Introduction

Peritoneal dialysis (PD) is one of the important renal replacement therapies in patients with end-stage renal disease (ESRD), and the number of ESRD patients who choose PD as their first renal replacement therapy modality is increasing [1]. However, patients undergoing PD treatment have a higher mortality risk compared with the general population, largely owing to cardiovascular disease (CVD), which accounts for nearly 40% [2]. Previous studies have found that various risk factors independently contribute to the cardiovascular (CV) mortality in PD patients, including pulmonary hypertension [3], triglyceride to high-density lipoprotein cholesterol ratio [4], high peritoneal dialysate glucose concentration [5], and inflammation [6].

Platelet plays a central role in the pathophysiology of hemostasis and thrombosis, leading to atherosclerotic CVD and other ischemic diseases [7, 8]. Platelets are rapidly recruited in the injured vascular endothelium from flowing blood and then engage collagen in the vessel wall to adhere and aggregate on the damaged vascular endothelial cells, which results in hemostasis and thrombosis [9]. It had been demonstrated that larger platelet contains more prothrombotic cytokines and expresses more adhesion receptors [10]. Therefore, the mean platelet volume (MPV), reflecting the average size of platelet, is considered as an indicator of platelet function and activation [11]. Previous studies indicated that elevated MPV was associated with higher mortality in hemodialysis patients but not in PD patients [12, 13]. Moreover, it was reported that there was a negative correlation between MPV and platelet count in normal population [14], which indicated that MPV and platelet count could be interpreted as a ratio rather than separate variable.

Recently, MPV/platelet count ratio (MPV/PC) was demonstrated as a predictor of mortality in some populations, such as CVD [15–17] and non-small-cell lung cancer [18]. In patients with non-ST-segment elevation myocardial infarction, Azab et al. [15] found that patients in the highest and lowest MPV/PC tertiles had higher mortality compared to those in the middle MPV/PC tertile, and they also found that the MPV/PC ratio was superior to the MPV alone in predicting long-term mortality. Inagaki et al. [18] showed that decreased MPV/PC ratio was independently associated with an increased mortality in patients with non-small-cell lung cancer. In addition, it was reported that elevated longitudinal MPV/PC was an independent risk factor for vascular access failure in hemodialysis patients [19]. However, little attention has been paid to the association between MPV/PC and mortality in PD patients. Therefore, we aimed to evaluate the association between MPV/PC and the risk of all-cause and CV mortality in PD patients.

## 2. Materials and Methods

### 2.1. Study Population

We designed a single-center, retrospective cohort study which included all patients treated with PD from January 1, 2006, to December 31, 2013 at the First Affiliated Hospital of Sun Yat-sen University. Patients who were aged ≥ 18 years at the initiation of PD, received PD therapy for at least 3 months, and had catheter insertion in our PD center were enrolled. The exclusion criteria for this study were patients who received hemodialysis (HD) for more than 90 days, transferred from failed renal transplantation, had a history of malignant tumors, or lacked of baseline MPV/PC values. This study was carried out according to the ethical principles of the Declaration of Helsinki and was approved by the Research Ethics Committee of The First Affiliated Hospital, Sun Yat-sen University. All participants provided written informed consent.

### 2.2. Data Collection

Baseline demographic and clinical data such as age, gender, body mass index (BMI), primary disease, comorbid conditions, and Charlson comorbidity index (CCI) were obtained from patients' medical records. Laboratory parameters at the initiation of PD included hemoglobin, platelet, MPV, serum albumin, serum creatinine, blood urea nitrogen (BUN), serum uric acid, total cholesterol, triglycerides, high-sensitivity C-reactive protein (hs-CRP), residual renal function (RRF), and total urea clearance index (Kt/V). All data were obtained within 3 months after the start of PD therapy. RRF was measured as mean values of creatinine clearance and urea clearance and corrected for body surface area [20]. CVD was defined as a history of heart failure, coronary heart disease, cerebrovascular event, or peripheral vascular disease.

### 2.3. Outcomes

In the present study, we hypothesized that MPV/PC level was an independent risk factor for all-cause and CV mortality in PD patients. The primary clinical outcome was all-cause mortality. The secondary outcome was CV mortality, which was defined as death due to myocardial ischemia or infarction, congestive heart failure, sudden cardiac arrest, and cerebrovascular disease [21]. Patients were followed up until death, transferring to HD or renal transplantation, moving to other PD centers, abandonment of treatment, loss to follow-up, or end of the study (December 31, 2018).

### 2.4. Statistical Analysis

The patients were categorized into four groups according to the quartiles of baseline MPV/PC levels. Results were presented as mean ± standard deviation (SD) for normal-distributed continuous variables or median (interquartile range, IQR) for skewed-distributed continuous data and frequencies and percentages for categorical variables. Baseline characteristics of four groups were compared using one-way analysis of variance (normal distribution) or Kruskal-Wallis tests (skewed distribution) for continuous variables and chi-square test for categorical variables. The correlations between MPV/PC levels and baseline clinical parameters were analyzed by Spearman correlation analysis. We used Kaplan-Meier method to assess the cumulative survival rate of PD patients and log-rank test to compare the differences of survival rate among four groups. Cox proportional hazards model was applied to explore the association between MPV/PC and mortality in PD patients. Univariate analysis was first examined followed by adjustment models. Covariates that were thought to be related to MPV/PC level or to have clinically significant influence on PD patients' survival were chosen for multivariate Cox regression models. The interaction of age and MPV/PC with mortality was also evaluated by the Cox regression model with adjustment of potential confounders. The results were expressed as hazard ratio (HR) and 95% confidence interval (CI). A *P* value less than 0.05 was considered to be statistically significant. Statistical analyses were conducted using SPSS version 24.0 (IBM SPSS, Chicago, IL, USA).

## 3. Results

### 3.1. Baseline Characteristics of Enrolled Participants

**Figure 1:**
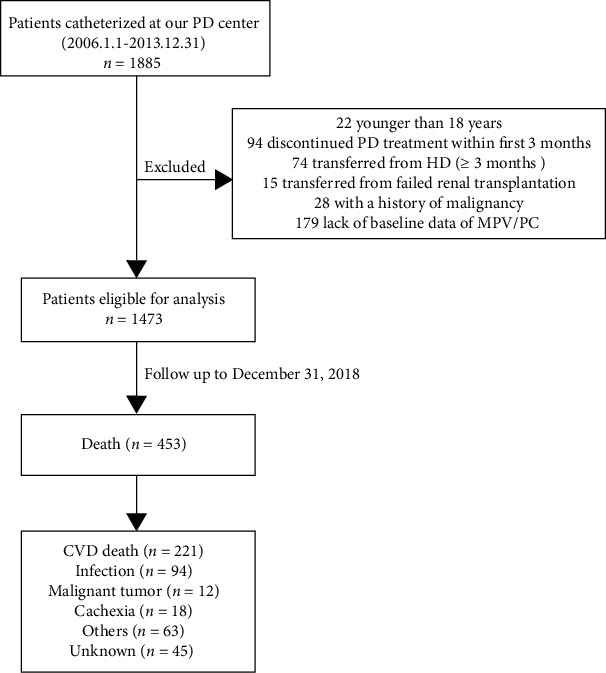
Flowchart of the participants in the study cohort. PD: peritoneal dialysis; HD: hemodialysis; MPV/PC: mean platelet volume to platelet count ratio; CVD: cardiovascular disease.

A total of 1885 ESRD patients catheterized at our PD center from January 1, 2006, to December 31, 2013, were included. According to the exclusion criteria, 412 patients were excluded in this study (Figure 1). Among the enrolled 1473 PD patients, the average age was 47.29 ± 15.28 years, 59.3% were male, and 38.4% had a history of CVD. The leading cause of ESRD was primary glomerulonephritis (60.1%), followed by diabetic nephropathy (22.3%). The baseline MPV/PC level was 0.052 ± 0.028. Patients were classified into four groups according to the quartiles of baseline MPV/PC levels. Baseline demographic and clinical characteristics of different groups are shown in Table 1. Compared with higher MPV/PC groups, patients with lower MPV/PC were older, were more likely to have a history of CVD, hypertension, and diabetes; had higher levels of BMI, CCI, total cholesterol, triglyceride, hs-CRP, and total Kt/V; and were more inclined to take antiplatelet drugs.

### 3.2. Correlation of MPV/PC with Relevant Parameters

The correlation analyses showed that MPV/PC negatively correlated with age (*r* = −0.126, *P* < 0.001), BMI (*r* = −0.100, *P* < 0.001), CCI (*r* = −0.100, *P* < 0.001), hemoglobin (*r* = −0.067, *P* = 0.011), hs-CRP (*r* = −0.091, *P* = 0.001), total cholesterol (*r* = −0.116, *P* < 0.001), triglyceride (*r* = −0.115, *P* < 0.001), RRF (*r* = −0.119, *P* < 0.001), and total Kt/V (*r* = −0.088, *P* = 0.001) (Table 2).

### 3.3. MPV/PC Levels and Mortality

The median follow-up time was 48.0 (21.9-82.2) months. By the end of the study, 453 (30.8%) patients died, 356 (24.2%) patients underwent renal transplantation, and 281 (19.1%) patients were transferred to hemodialysis. Of the 453 deaths, 221 (48.8%) deaths were caused by CVD, 94 (20.8%) deaths were caused by infective diseases, 12 (2.6%) deaths were due to malignant tumors, 18 (4.0%) deaths were due to cachexia, 63 (13.9%) deaths were due to other reasons, and 45 (9.9%) deaths were with unknown reason.

**Figure 2:**
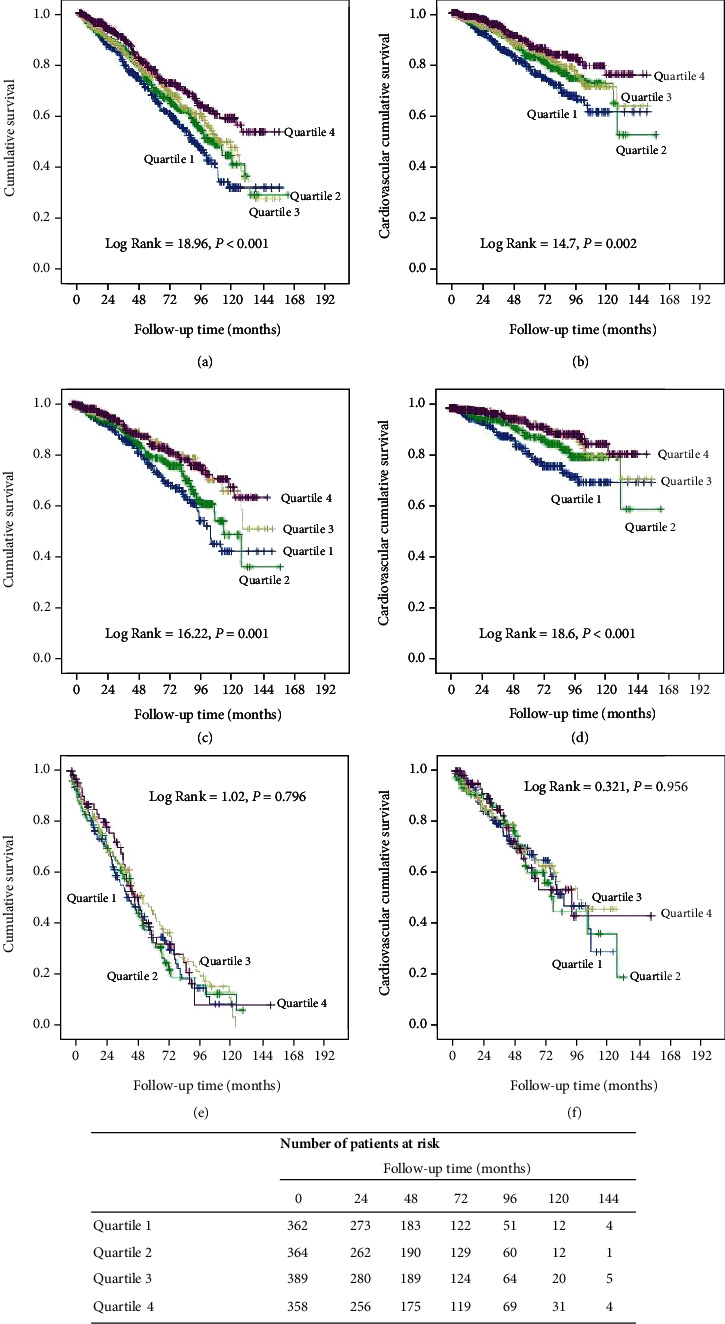
Kaplan-Meier survival curves for patients with different levels of MPV/PC and stratified by age. Cumulative survival curves for (a, b) overall population, (c, d) age < 60 years, and (e, f) age ≥ 60 years.

Figure 2 showed the cumulative survival rates which were analyzed by Kaplan-Meier survival curve. The cumulative survival rates at the end of 1, 3, 5, and 10 years were 87.5%, 74.6%, 60.4%, and 30.5% in quartile 1; 90.5%, 80.0%, 66.7%, and 35.4% in quartile 2; 90.9%, 80.5%, 69.1%, and 36.3% in quartile 3; and 94.4%, 82.4%, 72.7%, and 54.4% in quartile 4, respectively. Compared with higher MPV/PC levels, the survival rate was significantly decreased in the lower MPV/PC level (log − rank = 18.96, *P* < 0.001) (Figure 2(a)). Cardiovascular survival rates at the end of 1, 3, 5, and 10 years were 92.3%, 84.1%, 75.1%, and 60.2% in quartile 1; 95.3%, 90.8%, 82.1%, and 64.9% in quartile 2; 95.6%, 90.4%, 83.4%, and 70.8% in quartile 3; and 97.8%, 91.5%, 85.9%, and 76.3% in quartile 4, respectively. Similarly, patients with higher MPV/PC levels had lower cardiovascular survival rate among the groups (log − rank = 14.7, *P* = 0.002) (Figure 2(b)).

The association between MPV/PC with all-cause and CV mortality is shown in Table 3. The univariate Cox regression model showed that, compared with quartile 1, the risk of all-cause and CV mortality in patients with the highest MPV/PC level was decreased (HR = 0.55, 95%CI = 0.40 − 0.74, *P* < 0.001; HR = 0.47, 95%CI = 0.31 − 0.73, *P* = 0.001, respectively). The association between baseline MPV/PC ratio and mortality had similar results when MPV/PC was examined as a continuous variable. However, after adjusting for age, gender, BMI, CCI, hemoglobin, albumin, total cholesterol, triglyceride, hs-CRP, total Kt/V, RRF, and the use of antiplatelet drugs, the association between MPV/PC with all-cause and CV mortality was not significant (HR = 0.86, 95%CI = 0.63 − 1.19, *P* = 0.366; HR = 0.83, 95%CI = 0.53 − 1.30, *P* = 0.412, respectively).

The relationship between baseline MPV/PC with all-cause and CV mortality in subgroup analysis stratified by age was further performed. Kaplan-Meier survival analysis showed that the overall survival rate and cardiovascular survival rate were significantly decreased in the lower MPV/PC level in patients with age < 60 years (log − rank = 16.22, *P* = 0.001; log − rank = 18.6, *P* < 0.001, respectively) (Figures 2(c) and 2(d)), but not in those with age ≥ 60 years (log − rank = 1.02, *P* = 0.796; log − rank = 0.321, *P* = 0.956, respectively) (Figures 2(e) and 2(f)). As shown in Tables 4 and 5, an interaction effect was found between age and MPV/PC level on all-cause mortality (*P* = 0.009). In PD patients aged < 60 years, the result demonstrated that the fourth quartile of MPV/PC level was related to a decreased risk for all-cause and CV mortality compared with quartile 1 even after adjusting for covariates (HR = 0.62, 95%CI = 0.40 − 0.96, *P* = 0.032; HR = 0.49, 95%CI = 0.26 − 0.93, *P* = 0.029, respectively). However, this association did not exist in patients aged ≥ 60 years (HR = 1.37, 95%CI = 0.84 − 2.22, *P* = 0.208; HR = 1.50, 95%CI = 0.77 − 2.92, *P* = 0.237, respectively).

## 4. Discussion

In this retrospective cohort study, we found that there was a significant age-specific interaction between MPV/PC level and all-cause mortality, while the interaction effect between age and MPV/PC level on CV mortality was not significant. Our results also showed that a decreased MPV/PC level was an independent risk factor for all-cause and CV mortality in PD patients aged less than 60 years. However, no association was observed between MPV/PC levels and mortality in PD patients aged 60 years or older.

Previous studies have shown that the levels of some platelet indices, including platelet count, plateletcrit, MPV, and platelet distribution width, were associated with mortality in patients undergoing dialysis [12, 13]. However, the association between platelet indices with mortality in dialysis patients was controversial. Peng et al. found that higher platelet levels, but not MPV, were significantly associated with higher CV mortality in PD patients [13]. Another study demonstrated that the risk of all-cause and CV mortality was increased in patients with lower MPV levels, and platelet count was not associated with patient's mortality in PD population [22]. In addition, elevated MPV level can predict higher mortality in incident hemodialysis patients [12]. Therefore, platelet and MPV alone may not be a reliable predictor of mortality in PD patients. Emerging evidences suggested that a combination of platelet and MPV may be more clinically significant than platelet or MPV alone in various diseases, including CVD, cancer, and kidney diseases [17, 22–25]. Moreover, time-averaged MPV/PC level was an independent risk factor for vascular access failure in patients undergoing hemodialysis [19]. Another study including 338 PD patients revealed that elevated MPV/PC was significantly associated with lower all-cause and CV mortality in PD patients [22]. However, in the present study, we found that higher MPV/PC levels were associated with lower risk of all-cause and CV mortality in PD patients aged less than 60 years rather than in those aged 60 years or older. These inconsistencies might be due to different follow-up times, sample sizes, and adjusted covariates.

In addition to being important in hemostasis and thrombosis, platelet is also involved in modulating inflammatory reactions [26]. It has been reported that platelet was positively correlated with hs-CRP and neutrophil-to-lymphocyte ratio (NLR), which are well-established markers of inflammation [13]. The platelet could release proinflammatory cytokines and inflammatory mediators, which can enhance leukocyte recruitment and lead to the further release of inflammatory cytokines [27]. Moreover, MPV is associated with different inflammatory conditions [28]. Some studies have indicated that decreased MPV can be used as an inflammation marker for certain disease, such as ulcerative colitis [29], systemic lupus erythematosus [30, 31], and rheumatoid arthritis [32]. Although MPV is elevated in the early stage of inflammation, a decreased MPV could be observed in patients with persistent inflammation [33]. In summary, elevated platelet and decreased MPV are both associated with inflammatory conditions, and thus, low MPV/PC may be a useful marker of inflammation. It had been demonstrated that MPV/PC was negatively correlated with NLR [22]. In the present study, we also found that MPV/PC was negatively associated with hs-CRP. All these results indicated that decreased MPV/PC could reflect the inflammatory state of PD patients.

Numerous studies showed that chronic inflammation was highly prevalent in patients with chronic kidney disease, especially in ESRD [34]. In addition, chronic inflammation was independently associated with high risk of mortality in dialysis patients [35, 36]. Most well-established markers of inflammation, such as hs-CRP and NLR, are proved to be independent risk factors for mortality in PD patients [37–39]. In the current research, we have found that decreased MPV/PC may be a useful marker of inflammation and was associated with a higher risk of all-cause mortality in PD patients aged less than 60 years. Therefore, MPV/PC can be used as an indicator for monitoring the prognosis of PD patients. Compared with other inflammatory markers, MPV/PC is routinely performed on admission and convenient to obtain; therefore, it is more feasible to be a therapeutic target to improve the prognosis and survival rate in PD patients.

Our study found an interaction between age and MPV/PC level, and decreased MPV/PC was associated with all-cause and CV mortality only in PD patients aged less than 60 years. Previous studies have shown that platelet reactivity increases with age [40], while platelet count decreases with advanced age [41]. In addition, MPV also increases with age [42]. These age-related alterations in platelet function may contribute to the increased incidence of thrombotic events [43]. Therefore, a possible explanation for why decreased MPV/PC is not a risk factor for mortality among older PD patients might due to that older patients are at a higher risk of thromboembolism, which may attenuate the impact of MPV/PC on prognosis in PD patients. Meanwhile, older PD patients are more likely to have PD-related comorbidities, like peritonitis, and more likely to develop malnutrition [44, 45]. All these factors may interfere with the effect of MPV/PC on mortality in PD patients. Nevertheless, the mechanism of the relationship between decreased MPV/PC and higher risk of mortality among younger PD patients is still unknown and needs to be further studied.

Our study had several limitations. First, due to the observational nature, we cannot draw causal association between MPV/PC levels and mortality in PD patients. Second, the nature of single-center study may cause the selection bias. However, the relatively large number of enrolled patients could make our results more reliable. Finally, we examined only baseline levels of MPV/PC rather than considering the change of it, and using longitudinal data would be more realistic.

## 5. Conclusions

In conclusion, our study showed that MPV/PC level was negatively correlated with the inflammatory indicator hs-CRP, and decreased MPV/PC was independently associated with higher risk of all-cause and CV mortality in PD patients aged less than 60 years, which suggested that it is necessary to monitor MPV/PC levels in PD patients, especially in younger ones.

## Figures and Tables

**Table 1:** Baseline characteristics of patients stratified by MPV/PC value.

Variables	MPV/PC					*P* value
	Total (*n* = 1473)	<0.035 (*n* = 362)	0.035-0.045 (*n* = 364)	0.046-0.060 (*n* = 389)	>0.060 (*n* = 358)	
MPV/PC	0.052 ± 0.028	0.029 ± 0.004	0.040 ± 0.003	0.052 ± 0.004	0.087 ± 0.034	
Age (years)	47.29 ± 15.28	49.86 ± 14.51	47.1 ± 15.12	48.17 ± 16.04	43.92 ± 14.76	<0.001
Male gender (%)	873 (59.3%)	204 (56.4%)	228 (62.6%)	239 (61.4%)	202 (56.4%)	0.176
BMI (kg/m^2^)	21.60 ± 3.14	22.05 ± 3.36	21.79 ± 3.25	21.61 ± 3.11	20.94 ± 2.72	<0.001
Primary disease						<0.001
Glomerulonephritis (%)	886 (60.1%)	180 (49.7%)	212 (58.2%)	233 (59.9%)	261 (72.9%)	
Diabetic nephropathy (%)	328 (22.3%)	108 (29.8%)	94 (25.8%)	84 (21.6%)	42 (11.7%)	
Hypertensive nephropathy (%)	107 (7.3%)	32 (8.8%)	27 (7.4%)	28 (7.2%)	20 (5.6%)	
Others (%)	152 (10.3%)	42 (11.6%)	31 (8.5%)	44 (11.3%)	35 (9.8%)	
Hypertension (%)	291 (19.8%)	89 (24.6%)	75 (20.6%)	76 (19.5%)	51 (14.2%)	0.006
Diabetes (%)	375 (25.5%)	120 (33.1%)	104 (28.6%)	95 (24.4%)	56 (15.6%)	<0.001
CVD (%)	565 (38.4%)	169 (46.8%)	138 (37.9%)	147 (37.8%)	111 (31.1%)	<0.001
CCI	3.57 ± 1.84	3.81 ± 1.88	3.62 ± 1.88	3.61 ± 1.83	3.23 ± 1.74	<0.001
Hemoglobin (g/L)	100.04 ± 21.77	99.13 ± 21.33	102.87 ± 20.61	100.66 ± 22.51	97.42 ± 22.23	0.006
Platelet (10^9^/L)	218.75 ± 81.06	324.66 ± 58.94	237.71 ± 24.20	188.06 ± 21.62	125.72 ± 32.10	<0.001
MPV (fL)	9.59 ± 0.89	9.24 ± 0.67	9.38 ± 0.72	9.65 ± 0.81	10.10 ± 1.05	<0.001
Serum albumin (g/L)	37.02 ± 5.09	36.63 ± 5.09	37.25 ± 5.09	37.11 ± 5.05	37.06 ± 5.12	0.392
Total cholesterol (mmol/L)	4.98 ± 1.33	5.25 ± 1.44	5.07 ± 1.29	4.85 ± 1.29	4.73 ± 1.22	<0.001
Triglyceride (mmol/L)	1.36 (0.96-1.96)	1.48 (1.01-2.28)	1.42 (1.01-2.00)	1.35 (0.98-2.06)	1.28 (0.94-1.76)	< 0.001
Uric acid (*μ*mol/L)	432.55 ± 96.98	428.45 ± 98.66	426.79 ± 95.45	438.26 ± 95.39	436.34 ± 98.42	0.281
hs-CRP (mg/L)	1.80 (0.70-6.14)	3.02 (0.91-9.29)	1.86 (0.66-5.78)	1.55 (0.54-5.25)	1.22 (0.55-3.75)	< 0.001
Urea nitrogen (mmol/L)	16.6 (13.2-21.6)	16.1 (12.5-20.8)	16.6 (13.3-21.6)	16.1 (12.8-20.3)	16.6 (13.6-21.4)	0.014
Creatinine (*μ*mol/L)	717 (571-928)	676 (550-850)	683 (562-895)	727 (554-930)	773 (614-958)	<0.001
RRF (mL/min/1.73 m^2^)	3.21 (1.84-5.03)	3.34 (1.91-5.51)	3.38 (2.16-5.31)	3.33 (2.05-5.06)	2.70 (1.48-4.04)	<0.001
Total Kt/V	2.45 ± 0.72	2.52 ± 0.72	2.45 ± 0.64	2.48 ± 0.73	2.35 ± 0.79	0.016
Antiplatelet drugs	140 (9.5%)	49 (13.5%)	36 (9.9%)	32 (8.2%)	23 (6.4%)	0.009

MPV/PC: mean platelet volume to platelet count ratio; BMI: body mass index; CVD: cardiovascular disease; CCI: Charlson comorbidity index; MPV: mean platelet volume; hs-CRP: high sensitive C-reactive protein; RRF: residual renal function; Kt/V: urea clearance index.

**Table 2:** Relationship of MPV/PC with relevant parameters.

Variable	*r*	*P* value
Age (years)	-0.126	<0.001
BMI (kg/m^2^)	-0.100	<0.001
CCI	-0.100	<0.001
Hemoglobin (g/L)	-0.067	0.011
hs-CRP (mg/L)	-0.091	0.001
Albumin (g/L)	-0.022	0.391
Total cholesterol (mmol/L)	-0.116	<0.001
Triglyceride (mmol/L)	-0.115	<0.001
Uric acid (mmol/L)	0.025	0.344
RRF (mL/min/1.73 m^2^)	-0.119	<0.001
Total Kt/V	-0.088	0.001

MPV/PC: mean platelet volume to platelet count ratio; BMI: body mass index; CCI: Charlson comorbidity index; hs-CRP: high sensitive C-reactive protein; RRF: residual renal function; Kt/V: urea clearance index.

**Table 3:** The association of MPV/PC with all-cause and CV mortality in PD patients.

	Model 1^a^		Model 2^b^		Model 3^c^	
	HR (95% CI)	*P* value	HR (95% CI)	*P* value	HR (95% CI)	*P* value
*All-cause mortality*						
Continuous MPV/PC^d^	0.91 (0.87-0.96)	<0.001	0.96 (0.91-1.00)	0.058	0.97 (0.92-1.02)	0.177
Quartile 1	1.0		1.0		1.0	
Quartile 2	0.78 (0.60-1.02)	0.074	0.89 (0.68-1.17)	0.412	0.97 (0.73-1.28)	0.814
Quartile 3	0.76 (0.57-1.00)	0.050	0.73 (0.55-0.96)	0.026	0.76 (0.57-1.02)	0.063
Quartile 4	0.55 (0.40-0.74)	<0.001	0.76 (0.56-1.04)	0.085	0.86 (0.63-1.19)	0.366
*P* for trend		0.002		0.109		0.256

*CV mortality*						
Continuous MPV/PC^d^	0.90 (0.83-0.96)	0.002	0.94 (0.88-1.01)	0.085	0.96 (0.90-1.03)	0.277
Quartile 1	1.0		1.0		1.0	
Quartile 2	0.68 (0.46-0.99)	0.044	0.78 (0.54-1.15)	0.210	0.90 (0.61-1.33)	0.591
Quartile 3	0.68 (0.46-1.00)	0.048	0.66 (0.45-0.97)	0.034	0.76 (0.51-1.13)	0.173
Quartile 4	0.47 (0.31-0.73)	0.001	0.68 (0.44-1.05)	0.083	0.83 (0.53-1.30)	0.412
*P* for trend		0.006		0.128		0.571

MPV/PC: mean platelet volume to platelet count ratio; PD: peritoneal dialysis; CV: cardiovascular; HR: hazard ratio; CI: confidence interval. ^a^Unadjusted model. ^b^Adjusted for age, gender, and body mass index. ^c^Adjusted for model 2 covariates and Charlson comorbidity index, hemoglobin, albumin, total cholesterol, triglycerides, high-sensitivity C-reactive protein, total Kt/V, residual renal function, and the use of antiplatelet drugs. ^d^Per 0.01 higher MPV/PC.

**Table 4:** The association of MPV/PC with all-cause mortality stratified by age in PD patients.

	Model 1^a^		Model 2^b^		Model 3^c^	
	HR (95% CI)	*P* value	HR (95% CI)	*P* value	HR (95% CI)	*P* value
Interaction analysis (MPV/PC × age)^d^	*P* for interaction < 0.001		*P* for interaction < 0.001		*P* for interaction = 0.009	

Age < 60 (*n* = 1118)						
Continuous MPV/PC^e^	0.92 (0.86-0.98)	0.010	0.93 (0.87-0.99)	0.021	0.94 (0.88-1.01)	0.071
Quartile 1	1.0		1.0		1.0	
Quartile 2	0.79 (0.55-1.15)	0.218	0.80 (0.55-1.16)	0.236	0.88 (0.60-1.29)	0.521
Quartile 3	0.54 (0.35-0.83)	0.005	0.54 (0.35-0.84)	0.006	0.60 (0.38-0.94)	0.026
Quartile 4	0.51 (0.34-0.78)	0.002	0.55 (0.36-0.84)	0.005	0.62 (0.40-0.96)	0.032
*P* for trend		0.003		0.008		0.052

Age ≥ 60 (*n* = 355)						
Continuous MPV/PC^e^	0.96 (0.89-1.04)	0.331	0.97 (0.90-1.05)	0.418	1.01 (0.94-1.09)	0.742
Quartile 1	1.0		1.0		1.0	
Quartile 2	0.93 (0.62-1.39)	0.718	0.95 (0.63-1.44)	0.821	1.18 (0.77-1.81)	0.450
Quartile 3	0.91 (0.63-1.31)	0.603	0.89 (0.61-1.29)	0.535	0.94 (0.64-1.40)	0.769
Quartile 4	0.91 (0.58-1.43)	0.681	0.94 (0.59-1.48)	0.775	1.37 (0.84-2.22)	0.208
*P* for trend		0.955		0.942		0.422

MPV/PC: mean platelet volume to platelet count ratio; PD: peritoneal dialysis; CV: cardiovascular; HR: hazard ratio; CI: confidence interval. ^a^Unadjusted model. ^b^Adjusted for gender and body mass index. ^c^Adjusted for model 2 covariates and Charlson comorbidity index, hemoglobin, albumin, total cholesterol, triglycerides, high-sensitivity C-reactive protein, total Kt/V, residual renal function, and the use of antiplatelet drugs. ^d^Continuous MPV/PC × continuous age. ^e^Per 0.01 higher MPV/PC.

**Table 5:** The association of MPV/PC with cardiovascular mortality stratified by age in PD patients.

	Model 1^a^		Model 2^b^		Model 3^c^	
	HR (95% CI)	*P* value	HR (95% CI)	*P* value	HR (95% CI)	*P* value
Interaction analysis (MPV/PC × age)^d^	*P* for interaction < 0.001		*P* for interaction < 0.001		*P* for interaction = 0.140	

Age < 60 (*n* = 1118)						
Continuous MPV/PC^e^	0.88 (0.80-0.98)	0.015	0.90 (0.81-0.99)	0.033	0.92 (0.84-1.02)	0.102
Quartile 1	1.0		1.0		1.0	
Quartile 2	0.64 (0.39-1.06)	0.085	0.65 (0.39-1.07)	0.092	0.77 (0.45-1.30)	0.324
Quartile 3	0.46 (0.25-0.82)	0.009	0.46 (0.26-0.83)	0.010	0.55 (0.30-1.01)	0.053
Quartile 4	0.37 (0.20-0.67)	0.001	0.40 (0.22-0.74)	0.003	0.49 (0.26-0.93)	0.029
P for trend		0.003		0.008		0.089

Age ≥ 60 (*n* = 355)						
Continuous MPV/PC^e^	0.96 (0.87-1.07)	0.471	0.97 (0.87-1.08)	0.577	1.01 (0.91-1.13)	0.802
Quartile 1	1.0		1.0		1.0	
Quartile 2	0.85 (0.47-1.53)	0.591	0.90 (0.50-1.62)	0.724	1.16 (0.63-2.13)	0.643
Quartile 3	0.88 (0.52-1.50)	0.648	0.87 (0.51-1.47)	0.600	1.01 (0.57-1.76)	0.986
Quartile 4	0.96 (0.51-1.81)	0.905	1.01 (0.54-1.91)	0.968	1.50 (0.77-2.92)	0.237
*P* for trend		0.945		0.940		0.640

MPV/PC: mean platelet volume to platelet count ratio; PD: peritoneal dialysis; CV: cardiovascular; HR: hazard ratio; CI: confidence interval. ^a^Unadjusted model. ^b^Adjusted for gender and body mass index. ^c^Adjusted for model 2 covariates and Charlson comorbidity index, hemoglobin, albumin, total cholesterol, triglycerides, high-sensitivity C-reactive protein, total Kt/V, residual renal function, and the use of antiplatelet drugs. ^d^Continuous MPV/PC × continuous age. ^e^Per 0.01 higher MPV/PC.

## Data Availability

The clinical data used in the present study could be available from the corresponding author.
